# Motivations and expectations driving community participation in entomological research projects: Target Malaria as a case study in Bana, Western Burkina Faso

**DOI:** 10.1186/s12936-020-03277-7

**Published:** 2020-06-05

**Authors:** Nourou Barry, Patrice Toé, Lea Pare Toe, Javier Lezaun, Mouhamed Drabo, Roch K. Dabiré, Abdoulaye Diabate

**Affiliations:** 1 Institut de Recherche en Sciences de la Santé/Centre Muraz, Bobo-Dioulasso, Burkina Faso; 2 Université Nazi BONI, Bobo-Dioulasso, Burkina Faso; 3 Institute for Science, Innovation and Society, School of Anthropology and Museum Ethnography, University of Oxford, Oxford, UK; 4 Department of Life Sciences, Imperial College of London, London, UK

**Keywords:** Public engagement, Entomological research activities, Malaria, Target Malaria, Burkina Faso

## Abstract

**Background:**

Most field entomology research projects require active participation by local community members. Since 2012, Target Malaria, a not-for-profit research consortium, has been working with residents in the village of Bana, in Western Burkina Faso, in various studies involving mosquito collections, releases and recaptures. The long-term goal of this work is to develop innovative solutions to combat malaria in Africa with the help of mosquito modification technologies. Since the start of the project, Bana residents have played an important role in research activities, yet the motivations and expectations that drive their participation remain under-investigated. This study examines the factors that motivate some members of the local community to contribute to the implementation of Target Malaria’s activities, and, more broadly, explores the reasons that animate citizen participation in entomological research work in malaria-endemic regions.

**Methods:**

A qualitative approach was used to survey the factors motivating members of the local community to assist in the implementation of Target Malaria’s entomological research activities in Bana. Eighty-five individual in-depth and semi-structured interviews were conducted, followed by three focus groups, one with youths who had participated in mosquito collections, and two with adult men and women from the village. All data collected were fully transcribed, processed, and subjected to thematic content analysis.

**Results:**

Data showed that the willingness of local community members to participate in entomological research activities was informed by a wide range of motivational factors. Although interviewees expressed their motivations under different semantic registers, the data showed a degree of consistency around five categories of motivation: (a) enhance domestic protection from mosquitoes and malaria, (b) contribute to a future world free of the disease, (c) acquire knowledge and skills, (d) earn financial compensation, and (e) gain social prestige for the village.

**Conclusion:**

These varying motivations reflect a set of differing personal and collective perceptions about the participation process, combining short and long-term, individual and collective motivations. Beyond the specific circumstances of this case, the study highlights the complex reasons that drive collective participation in entomological research and vector control activities. Detailed knowledge of community expectations should underpin any effort to mobilize local participation in field research activities.

## Background

Despite significant achievements in the control of malaria over the last two decades, progress in many regions seems to be coming to a halt [1]. Recent World Health Organization (WHO) World Malaria Reports [1, 2] indicate increasing rates of mortality and morbidity associated with malaria in several African countries, including Burkina Faso. The growth of mosquito resistance to mainstay insecticides constitutes perhaps the most serious threat to sustainable success with existing chemical control tools [3–5]. Different species of *Anopheles* mosquitoes show growing resistance to multiple pesticides [6, 7]. There is, as a result, a growing emphasis on the development of new tools and approaches, and on improving the integration of multiple malaria control strategies [8, 9]. Among the new approaches being considered is the modification of malaria vectors species to achieve population suppression or replacement [10, 11].

Target Malaria is a prominent example of this strategy. This collaborative research consortium seeks to develop new forms of genetic modification to suppress the ability of malaria mosquito vectors to reproduce [12, 13]. By using gene drive constructs, Target Malaria hopes to create strains of *Anopheles* mosquitoes able to transmit population-suppression traits to the vast majority of its offspring, thus allowing these traits to spread quickly through the population [14]. This technology could potentially become a key complementary tool in malaria control and eradication campaigns.

In Africa, Target Malaria operates in four countries: Mali, Uganda, Ghana and Burkina Faso. In Burkina Faso the consortium has been conducting research since 2012, including multiple entomological investigations to characterize local malaria vector species and define appropriate mosquito release programmes [15]. The research in Burkina Faso is led by researchers at the Institut de Recherche en Sciences de la Santé (IRSS), in Bobo-Dioulasso. The current paper focuses on work conducted during the period when baseline entomological research was carried out in the field, prior to the release of any genetically modified mosquitoes.

In Western Burkina Faso, baseline entomological research has been underway since July 2012 and communities in the area have been actively involved in a variety of investigational activities, including human landing catch sampling of mosquito populations, location and sampling of swarms, egg surveys, insecticide spray catches [16], and mark–release–recapture experiments [17, 18]. Residents also allowed access to their properties for entomological surveillance activities, including the positioning of mosquito traps.

Community participation is considered essential for attaining an effective control of disease vectors [19], and multiple studies attest to the importance of enrolling local residents in campaigns against mosquitoes in particular [20–24]. Unfortunately, these studies provide limited information on the factors that motivate local communities to become actively involved in these interventions, and the question of which particular expectations and considerations drive public participation remains as a result under-investigated. The scant information available is often limited to practical aspects of local participation [25–27] or to the ethical dimensions of individual and community recruitment [28]. Data pertaining to clinical research projects is of limited value in this case, as the expectation of an immediate and direct personal health benefit may in principle play a less prominent role in the willingness to participate in entomological research projects [29, 30].

Target Malaria thus offered a good opportunity to analyse in more detail the range of motivations driving local participation in entomological research activities. As the first initiative to explore the use of gene-drive modified mosquitoes for malaria control, Target Malaria has attracted a significant degree of attention. Its activities in Burkina Faso in particular have been criticized by national and international organizations opposed to the use of genetically modified organisms [31]. While there is undoubtedly a need for further research on the reasons that drive opposition to the use of gene drives and to mosquito modification technologies more generally, it is necessary to understand the motivations of those individuals and communities who choose to collaborate with a project of this kind. This knowledge is essential to the design of research programmes that can meet local expectations and mobilize community support over the long timespans required for robust entomological research and sustainable vector control interventions.

## Methods

### Study site

**Fig. 1:**
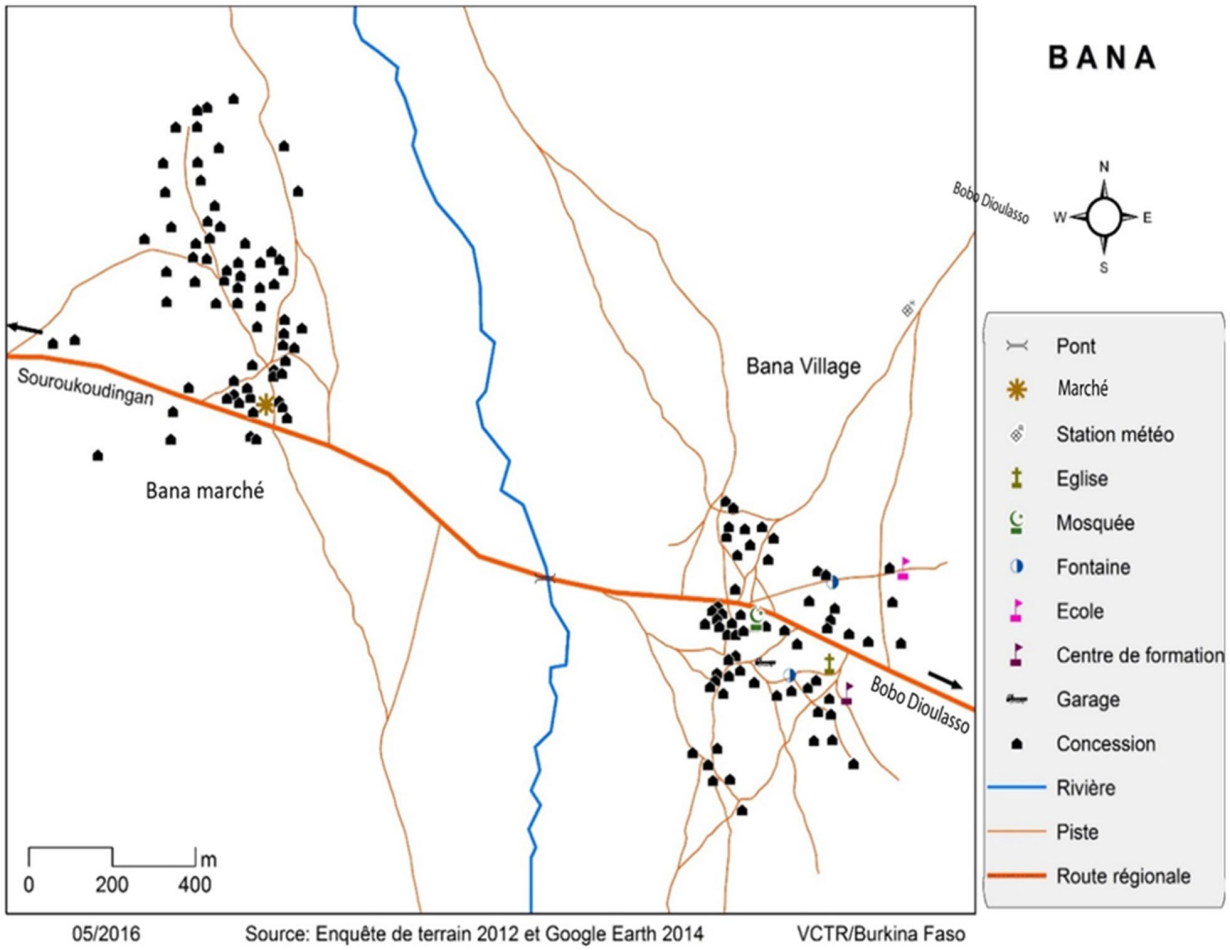
The location map of the village and the distribution of the area’s inhabitants

This study was conducted in the village of Bana, located in Western Burkina Faso, approximately 20 km from Bobo-Dioulasso (Fig. 1). In 2012, the population of Bana was estimated to be approximately 982 inhabitants (498 men and 484 women) [32]. Bana has two main inhabited areas, Bana Centre and Bana market, separated by a small river (usually dry during the dry season, from November to April). Bana Centre is the principal agglomeration and includes the village administration; Bana market is the economic hub of the village and the surrounding area, and hosts a busy weekly market. The whole village is a loose cluster of about 130 compounds (local census, October 2014). Each compound is typically a family unit consisting of between two and ten houses, generally made of mud. The largest ethnic groups are the Bobo, Dioula, Mossi, Fulani, and Sambla. The Bobo are the indigenous group in the area, and Dioula the most widely spoken language. Similar to most rural areas in Western Burkina Faso, Bana’s economy is essentially based on subsistence agriculture [33]. Vegetable growing is a particularly important economic activity, as it compensates for bad agricultural yields and is one of the few activities that can generate revenue during the 5–6 months of the dry season.

Malaria is endemic in the area. The density of *Anopheles* mosquitoes is typically highest in September–October and lowest around January–February. The extent of bed net coverage is high, although many of the nets are old [16, 17]. Virtually all the youths in the village do regular gardening as an income-generating activity, yet this activity is insufficient to meet their needs.

IRSS scientists began to carry out research in Bana on behalf of Target Malaria in 2012, after consultation with the village leaders. Baseline entomological activities included several methods of mosquito sampling, both in public space (peri-domestic areas, pathways, agricultural lands) and inside households. These were carried out in 20 so-called ‘fixed’ households, which were used for monthly collections, and 60 households that rotated monthly throughout the period of baseline entomological research.

Mark–release–recapture experiments, as well as egg collection and swarm sampling were carried out in open spaces, whereas sampling through human landing catches and spray catches were conducted indoors. Throughout the project, informed consent was obtained from village leaders for open field activities and from individual households for indoor research. Almost all the households in the village were involved in entomological research work of some kind, excepted two that opted out at the beginning of the programme (the main reason offered was the presence in the household of members who were allergic to insecticides). Several village youths were individually recruited to carry out human landing and spray catches, egg collections and swarm location and sampling.

In parallel to entomological research, the project team maintained constant communication with village residents through regular group interactions and dialogue sessions to discuss the project and its objectives. The objectives of the research were widely explained, including the link between sampling activities and the ultimate goal of developing genetically modified mosquitoes to suppress malaria vectors. No genetically modified mosquitoes were released in Bana, or indeed in any other location in Burkina Faso, during the period when the research presented in this paper was conducted. The only mosquito releases that took place were as part of mark–release–recapture studies and involved conventional mosquitoes [17]. The overarching goal of these deliberative activities was to obtain and sustain a ‘social license to operate’ in Bana [34–36]. In accordance with Target Malaria’s stakeholder engagement principles, local community members participated at the consultative, functional, interactive, and involvement levels, corresponding to the four levels of the participatory scale [37–39].

### Data collection

This was a qualitative study based on a combination of different social scientific methods, including participant observation of routine entomological work and interviews with community members. Emphasis was placed on understanding the local actors’ perception of their situations and the interactions that unfolded during different kinds of scientific work conducted in the village. As noted by Olivier de Sardan [40], anthropology produces reports and interpretations based directly on the local context and coherence of all available facts. The empirical surveys conducted as part of this research enabled us to gather experiential data and relatively detailed statements from local community members engaged in ongoing research activities in the area.

Data were collected between October 2017 to January 2018. Semi-structured interviews were conducted with 40 members of the 60 rotating households, focusing on the interviewee’s perception of the entomological research activities taking place in the village and his/her motivations to participate (or not participate) in them. More structured interviews were conducted with 45 Bana residents, 20 from the ‘fixed’ households and 25 youths recruited for mosquito collection activities. In total, we conducted 85 interviews. These interviews focused on the interviewees’ knowledge on Target Malaria activities and on the motivations that explained their participation.

Furthermore, three focus groups were conducted in the village. One included young volunteers involved in mosquito collections. The other two included adult residents who participated in entomological research activities in other ways (primarily by granting access to their premises for sampling activities). One of these focus group was conducted with female residents and a separate one with male residents.

Interviews and focus groups discussions were carried out in Dioula. Notes were taken during the semi-structured interviews, while the structured interviews and the focus group discussions were recorded and transcribed.

### Data processing and analysis

The data was processed and tabulated with the QDA Miner Lite software, a multi-dimensional database and qualitative data-processing software used to process large amounts of textual material. There was a thorough qualitative analysis of the data content [41] complying with key principles in qualitative social science research [42], and focusing on three key themes: perception of the overall research programme, community understanding of entomological research activities, and motivations driving participation in those activities. The interpretation of the results was primarily based on these data, but drew also on surveys and focus group discussions conducted on similar topics in other African countries [26, 43–46].

## Results

The data shows that the participation of local communities in the implementation of Target Malaria’s entomological research activities was informed by a wide range of motivations. Five key themes, however, emerged consistently in the interviews and focus group discussions, even if individual actors often expressed them through different semantic registers: (1) concern over mosquitoes and malaria and desire for better protection; (2) willingness to contribute to a future world free of malaria; (3) the value of acquiring knowledge and skills; (4) the significance of financial remuneration; (5) the impact of the research on the village’s social prestige.

### Concern over mosquitoes and malaria

**Fig. 2:**
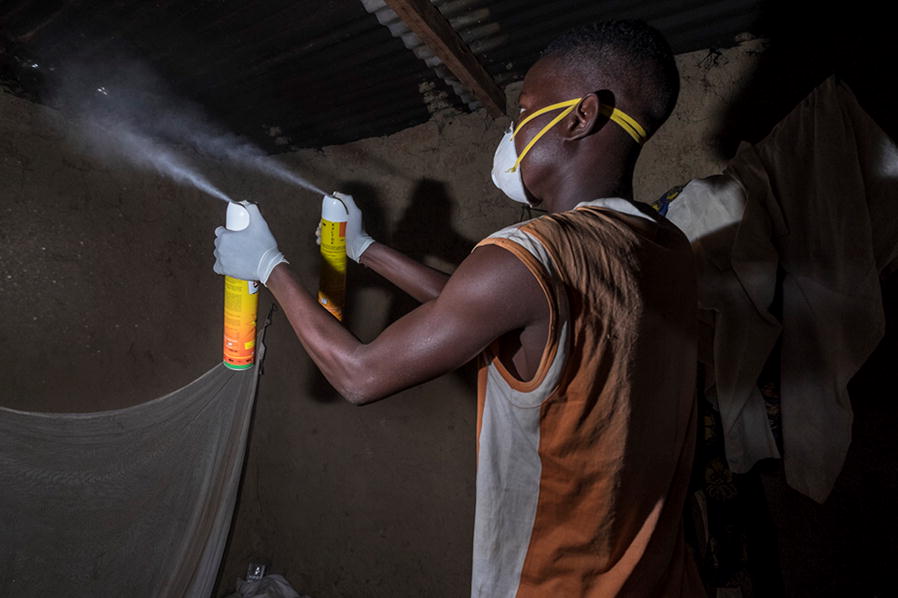
Spraying activity

The main reason why members of the local community in Bana participated in entomological research activities was the desire to protect themselves against mosquito bites and malaria. Fear of mosquitoes and of the diseases they transmit is an obvious concern in the area, and a vast majority of respondents drew a direct link between entomological research and better protection for themselves and their families. This motivation encompassed two overlapping considerations. On the one hand, residents valued the immediate protection provided by some research methods, particularly indoor spraying (Fig. 2). Indoor spray catches were used to assess the density of endophilic mosquitoes, as well as the proportion of blood-fed specimens and the degree of infectivity of different anopheline species. Most of the residents who granted access to their compounds for this kind of mosquito sampling valued the immediate protection it offered.

“*My husband and I have agreed for our home to be sprayed because we want to get rid of the mosquitoes in the house*” (35-year-old housewife, Bana).

“*Whenever they come to spray the house, they drive away a lot of mosquitoes. Again, we know that mosquitoes are not good. This is why I accepted to do the work. Because mosquitoes are not good.*” (women’s focus group, Bana).

Similar statements were gathered from other participants. Expressions such as “*We want to get rid of mosquitoes from our homes*”, “*We want good health*”, “*by spraying our homes, they chase out a lot of mosquitoes*” express both concern about mosquito-borne diseases and the assumption that some research activities produced a direct health benefit. In this regard, it is important to note that this assumption persisted despite repeated communications from the project team to the effect that entomological research activities, and sampling work in particular, were not intended as mosquito control interventions. In discussions with community members it was often noted that mosquito collection activities had a purely scientific purpose and did not offer lasting protection. It was emphasized that traditional protective measures, bed nets in particular, should be continuously used.

### *‘*Contribute to a better future’ and participate in ‘noble’ research

Another motivation mentioned by Bana residents to explain their participation in entomological research activities was their desire to ‘*contribute to a better future,*’ and specifically to a world without malaria. This theme emerged in a variety of interviews and focus group discussions, often in close relationship with the expectation of immediate personal or familial benefit. “*Mosquitoes are the ones which give us malaria; and to me, combating malaria is a good thing. As for me, I have children and I know that if malaria is controlled, one day my children will be free of this disease and that is good*” (participant in the women’s focus group, Bana).

Several interviewees considered Target Malaria an example of research that could provide a long-term solution for malaria.

“*As for me, I believe that we are contributing to a better future through our participation in the project. The mosquitoes which I catch myself, it’s as if I was working for myself or for my children. Even if I’m not alive tomorrow, at least I might have done something for tomorrow. For example, for those of us who are farmers, if you buy cattle and tomorrow you’re no more, your children can still work with the cattle. That is how this project is; we are searching for a solution to malaria to save tomorrow’s people*” (A 33-year-old mosquito-collecting youth, Bana).

Residents perceived that the various mosquito-collection activities were aimed at finding a future solution to malaria, and they believed that this was a noble objective and that by participating in the project they were doing “good” and contributing to improving the lives of future generations. The language in which this expectation was expressed often echoed messages put forward by the entomologists regarding the ultimate objective of their work (“contribute to a better future,” “serve tomorrow,” “noble research”).

### Useful knowledge and valuable technical skill

**Fig. 3:**
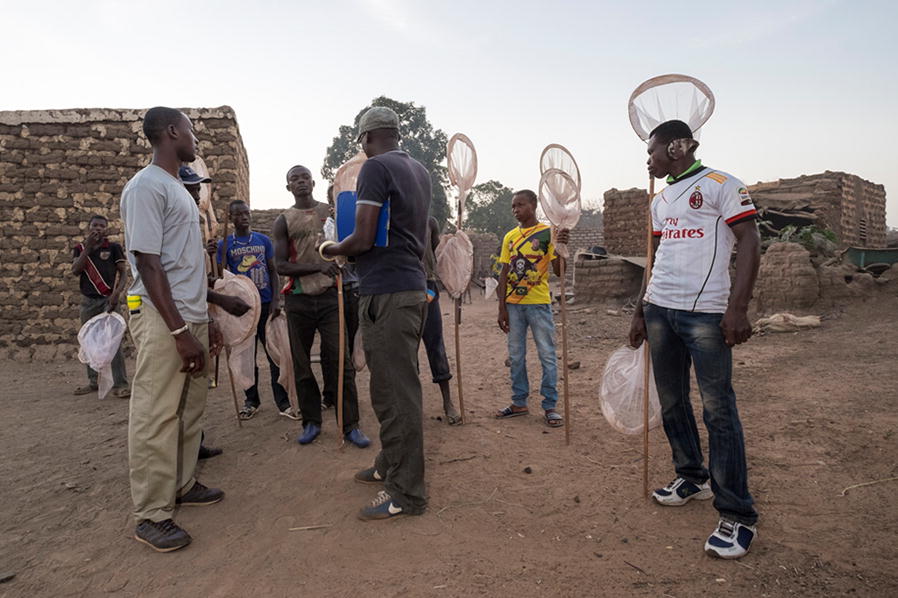
Session of preparation and setting up of mosquito collector

**Fig. 4:**
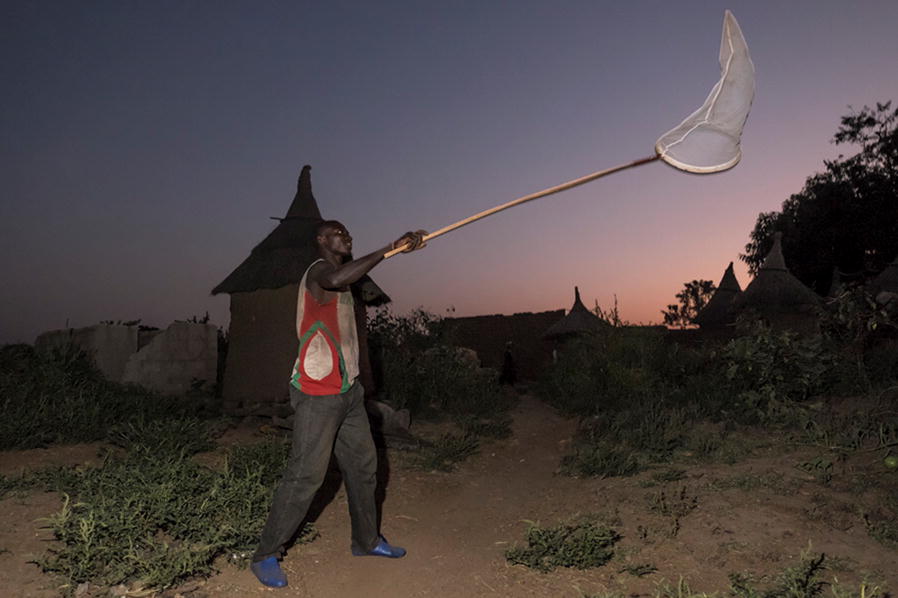
A collector of mosquito swarms in the village of Bana

Bana residents reported an appreciation of the opportunity to acquire new knowledge about mosquitoes and malaria transmission through their participation in Target Malaria, and in some cases were able to translate this knowledge into behaviours that might limit their exposure to mosquitoes. Several interviewees, particularly young mosquito collectors, considered their participation in entomological research work as an opportunity to learn practical new skills (Figs. 3, 4).

“*I came to do the work because it is a research that they are conducting. I want to learn this work. Knowing mosquitoes, knowing how to catch them in order to combat malaria*” (28-year-old mosquito collector, Bana).

As some participants in the focus group discussion said,

“*As for me, I wanted to learn how to capture a mosquito. I know that a mosquito is small. How are they able to catch it? This way, I can also learn how the mosquito transmits the disease*” (34-year-old mosquito collector, Bana).

“*Personally, it is an honour to help the project run smoothly. And I must admit that as mosquito collectors, we have learned a lot in this project. I never imagined I would catch mosquitoes one day because in my opinion it was absurd to do so. But with this project, I understood the need to catch mosquitoes and I also know all the techniques to catch them. This project teaches us how to protect ourselves from mosquito bites and the diseases they transmit*.” (38-year-old mosquito collector, Bana).

When asked what type of expertise they thought they were acquiring through their participation in the project, interviewees referred to a range knowledge and skills.

“*Thanks to the project, I learned how to catch mosquitoes. And then now I can differentiate between the kinds of mosquitoes according to whether they are male or female. I know that the male is the one who has a lot of hair on his mouth, he doesn’t bite. While the female has a small mouth and a long pointed beak and it is she who bites. In the evening, I can identify swarms of mosquitoes, whereas in the past I thought it was dust. As for the night, people may wonder how to catch these little bugs, we were shown this. For the third [activity], which is spraying, I learned how to pick them up on the sheet with a pair of pliers and put them in a box. I learned all this in the project*.” (24-year-old mosquito collector, Bana).

Another participant in the focus group with mosquito collectors emphasized

*“the fact that we always receive training from the project team on mosquito collection techniques before we start to practice it. I really enjoy it. Learning to capture mosquitoes is a good trade*” (32-year-old mosquito collector, Bana).

These comments show the extent to which some members of the community, particularly those who were recruited to conduct baseline entomological work (spray catches, swarm sampling, Human Landing Catch), perceived their participation as an opportunity to acquire valuable knowledge and skills. Furthermore, references to the importance of “doing the work” or “learning how to work” suggests that those who participated most directly in entomological research activities perceived their involvement as an opportunity to learn a “trade,” a set of skills that could be useful in future entomological research projects.

### Financial compensation

Target Malaria included a form of financial compensation for those who conducted work on behalf of the project. This included anyone in the village who voluntarily agreed to take part in the project’s entomological activities, either by allowing Target Malaria to use their household or by directly collecting mosquitoes on behalf of the project. Target Malaria insisted this was not a “remuneration” or “salary,” but rather a compensation for the time spent by local residents on research activities. Regardless of how this direct transfer was framed, it was often brought up in discussions with mosquito collectors.

“*Some of us, we are interested in this work because of the monetary reward attached to it. Something which helps us solve our petty financial problems. If someone asks me how is your work, I will tell them the mosquito*-*catching job is very good and that it is economically good to do this job. It is because of the money given after the work*.” (28-year-old mosquito collector, Bana).

The local expressions used by the volunteers to describe the financial compensation were very clear: “*Timinandiya*” (motivation or encouragement), “*Tͻnͻ*” (gain, interest, or profit) or “*Nusɔndiya*” (cheerful or being happy). These expressions, specially “*Tͻnͻ*”, referred to the financial resources provided by the project. The significance of financial compensation was also discussed in the focus groups with older residents. As the mother of a mosquito-collector youth said:

“*The village youths are our children. We know them very well. They offer to catch the mosquitoes because they gain something like financial benefit from it.*” (46-year-old housewife, mother of a mosquito-capturing youth, Bana).

### “Social prestige” and “village reputation”

Several statements from local residents, especially village leaders, highlight the role of prestige as one of the factors that motivated them to host the project.

“*As for me, I really understand the objectives of your project in our village… And I know that, thanks to this project, the name of the village will be projected into the limelight. And people all over the world will wonder where the village of Bana is located. The fact that such research is being conducted in this village is an immense motivation for us, for, in any case, it would bring immense social benefits to the village*” (46-year-old village leader, Bana).

For some residents, particularly those with a representative function, being associated with Target Malaria enhanced the village’s image, both at the national and international levels. This prestige was in some cases projected far into the future:

“*And, if in the meantime the project achieves the expected results and success in the work, even if we are not alive, people will say that research has been done in Bana. Bana’s name will be mentioned everywhere. And as a resident of the village, I will be very proud. That’s why I think it’s important to get involved*” (38-year-old men resident, Bana).

## Discussion

This study assessed the motivations that Bana residents articulate to explain their participation in entomological research activities sponsored by Target Malaria in their village. Motivation, as some authors [47–50] have reported, can be defined in this context as an intra-personal need or desire that stimulates a behaviour and gives it direction. It is something that energizes an individual to take action and that shapes her or his choices in goal-oriented behaviour [51]. Rooted in the interaction between the individuals and her or his work environment, motivations can be determined by a multiplicity of factors [48, 50]. Studies of voluntary service have shown that successful participation is driven by context-specific motivations [46, 52], and that these motivations can in some cases predict future willingness to participate.

The five categories described in this study highlight the range of motivational factors that explain willingness to participate in entomological research work. The interviewees emphasized their desire to join the fight against mosquitoes and malaria, to which all of them feel vulnerable. This vulnerability, and the attendant need to find more efficient ways of controlling mosquito-borne diseases, appears as the most important motivation influencing the decision to actively participate in Target Malaria project activities in Bana. The international dimension of Target Malaria, and the fact that it is trying to pioneer a novel method of malaria control, might explain the sense of pride reported by some respondents, and the degree of ‘prestige’ they associated with the village’s participation in the project.

The data also shows that residents value the knowledge and skills they acquire through participation. Several years of involvement with Target Malaria have substantially improved local understandings of malaria and malaria transmission, a crucial factor in successful disease control [53–55]. Mosquito collectors in particular list a wide variety of skills acquired since they joined the project in 2012, including how to capture mosquitoes, or how to identify different mosquito species [34, 35]. In some cases, they perceive entomological research as a long-term prospect, given the presence of an internationally recognized research group in Bobo-Dioulasso, and intend to capitalize on their experience to gain access to future projects in the area. Bana residents also value the opportunity to generate some extra income through the financial compensation offered by the project for participation in research activities.

This research contributes to an extensive literature on the complexities of research collaborations between institutions in the global North and the global South [53, 54]. While these collaborations are always vexed by large differentials of power, sometimes they create openings for the transfer of expertise and resources to disadvantaged locations in Africa and elsewhere [55–57].

Social scientific research into local participation in biomedical research projects in Africa has similarly showed a wide range of motivational factors. A research study conducted in Tanzania on the reasons given by mothers and guardians to explain their decision to enrol their children in malariometric trials, for example, demonstrated to the importance of gaining access to better healthcare services [58]. A similar survey conducted in Malawi found that a variety of direct financial or material benefits (soap, groundnut paste, orange juice, transportation money, tablecloths, mosquito nets, water basins, and iron tablets) was important in accounting for a high level of community participation [45, 59]. The data supports the relevance of material and transactional considerations, even though they are sometimes obscured and often difficult to discuss openly within the traditional ‘ethical’ framework that governs the organization of research efforts in under-resourced settings.

Yet apart from individual gain, this study suggests that a variety of more altruistic motivations are also at work, particularly the desire to contribute to a better future by participation in scientific activities, even when they offer only a delayed or indirect material benefit. In Bana, virtually all of the interviewees recognized that the full potential value of the research conducted under Target Malaria will only be achieved in the long term, if at all. Despite this awareness, however, and after many years of participation in research activities, local residents were able to articulate a set of long-term, prospective, often altruistic motivations for lending support to this project. The challenge for Target Malaria and other similar projects is to sustain the force of these motivational factors while communicating clearly that the benefits of any ensuing intervention will only materialize in the long term.

In adapting the conclusions of this study to other settings, it highlights the multidimensional set of motivations present in any individual or community recruited to participate in entomological research or vector control activities, and the importance of designing collaborations capable of offering a mix of immediate and long-term benefits, direct and indirect rewards for the communities hosting the work. This goes well beyond ensuring informed consent or providing clear explanations of the project and its objectives, and implies rooting the research in the everyday expectations and practical needs of the communities whose support sustains lasting field research efforts.

### Study limitations

The interviewees were aware that this research was sponsored by Target Malaria, and there was an obvious risk that they offered answers that they thought would appeal to the project team. To minimize this risk, the authors ensured that the data-gathering researcher (NB) was not involved in the main entomological research activities, which allowed him to immerse himself in the life of Bana over a significant period (3 months). This enabled him to develop a relationship of trust with a vast majority of the respondents and most likely reduced the scope of any potential bias.

## Conclusion

This study considered the factors motivating residents in a local community to join in entomological research activities for malaria vector control in Western Burkina Faso. A combination of qualitative social science methods was used to produce an in-depth understanding of the motivations and expectations driving residents in the village of Bana to participate in research activities sponsored by Target Malaria, an international consortium exploring the use of genetically modified mosquitoes to suppress malaria transmission. The data shows multiple reasons for engagement in entomological research activities. The desire to protect oneself and one’s family from mosquitoes and malaria, the chance to contribute to the development of a technology that might help rid the world of malaria, and the prestige that the village may acquire through its association with an international research endeavour were motivations shared by all virtually all participants. The opportunity to acquire new knowledge and skills and the prospect of a direct financial gain were also seen as important reasons, particularly by young residents recruited to do mosquito collections. Entomological field research activities require community support and involvement. Greater awareness of the motivations and expectations of community members is essential to the design of research programmes capable of obtaining robust and durable public support.

## Data Availability

The datasets generated and/or analysed during the current study are not publicly available, due to the fact that they are part of research project that is still ongoing. They are available from the corresponding author, however, on reasonable request.

## Abbreviations

IRSS: Institut de Recherche en Sciences de la Santé
WHO: World Health Organization
